# Promoting mental health and wellbeing as means to prevent disability: a review

**DOI:** 10.3389/fpubh.2024.1425535

**Published:** 2024-11-01

**Authors:** Yasir A. Alsamiri, Malik A. Hussain, Omar A. Alsamani, Abdulrahman A. Al bulayhi

**Affiliations:** ^1^Department of Education, Islamic University of Madinah, Madinah, Saudi Arabia; ^2^Department of Pathology, College of Medicine, University of Hail, Hail, Saudi Arabia; ^3^Department of Special Education, University of Hail, Hail, Saudi Arabia

**Keywords:** mental health, resilience, disability, coping skills, social support

## Abstract

**Background:**

Mental health is one of the key pillars of general welfare, and through its promotion, disability can be prevented.

**Objective:**

This research paper covers the field of literature that primarily addresses the increase of mental health and wellbeing aimed at preventing disability.

**Methodology:**

The analysis depends on secondary data that is acquired from different studies and reports to determine the link between mental health interventions and disability prevention. After screening, 50 articles eventually qualified for this narrative review.

**Results:**

The results indicate that prevention of mental health problems could reduce the risk of disability development by reducing underlying risk factors and, consequently, improving the quality of life. The study highlights the significance of early preventive interventions and support systems to curtail the onset of mental health disorders, which could later result in impairment. It does the same by advocating for multi-disciplinary approach that combines mental health promotion policies with existing disability prevention strategies to improve outcomes.

**Conclusion:**

The review points to mental health promotion as a preventative measure that can prevent disability and improve overall wellbeing. Through efforts to identify, prevent, and cure mental health problems, individuals can ensure optimal functioning and enjoy a better quality of life.

**Recommendation:**

This finding is indicative of a broader healthcare approach integrating mental health promotion that is aimed at diminishing the burden of disabilities and improving overall public health outcomes.

## 1 Introduction

Along with physical health and well-being, there is also a psychological and emotional aspect of life (1). Mental health along with physical health play crucial role in being healthy and helps avoid impairments. It is necessary to ensure that everybody, regardless of age or background, is well equipped to regard and maintain one’s mental health (2). Although mental health issues are being addressed by more and more people nowadays, there are still plenty of people who try to avoid getting help due to mental health problems stigma (3). This may lead to a variety of effects which may culminate in the development of mental disorders or may become more severe thereby impairing people.

The basis of this review study is to come up with the association between mental health, disability prevention, and overall wellness. Through encouraging mental health and wellbeing we would be preventing mental disorders that might lead to disability (4).

This review will discuss the role of mental health on disability prevention with involve the tendencies as well as the solutions and strategies that can promote mental health and social well-being. Through the understanding of mental health and disability links, we can design programs and interventions targeted at maintaining mental health and preventing disability (5).

Through this study, we explore the importance of mental health for the general health and wellbeing. Our aim is to create an environment in which physical and mental health are considered the same, leading to a better quality of life (6). One aim of the study is to promote a multilevel strategy for health and wellness through an addition to the rapidly growing literature on mental health, disability prevention, and wellbeing. We have described various aspects of mental health, its relationship with disability, different strategies to improve it, challenges in achieving this, and practically implementing it through policies and procedures. It is very important to understand all aspects in an integrated form. As most of the research articles and many reviews focus on one or few of these parameters, thus our article presents linkages among various aspects of the concept.

## 2 Literature review

The relationship between mental health and wellbeing and disability prevention has been established by multiple research studies that have demonstrated the link between mental health, wellbeing, and disability. Both physical and mental health are deeply linked to each other. This literature review provides an overview of the most important studies on this topic.

The study by Weare (7) used a nationwide population of adults to explore the relationship between mental health and disability. The study showed that the people with better mental health and wellness had fewer levels of disability and more chances of recovery from severe diseases that could have left them permanently disabled. This means that mental health and wellness are in fact the key to prevent any physical impairment. Thus, mental health and strength contributes to the lifestyle measures to ensure a healthy body and preventing it from harms.

On the other hand, another study by Blair et al. (8) investigated the connection between mental health and impairment. Reviewing the evidence shows a direct relationship between mental health problems and disability, with depression and anxiety being significantly connected to disability outcomes. The study underlined the significance of the early intervention and support for people suffering from mental health disorders for delaying the onset of disability. Moreover, Thieme et al.’s (9) study investigated the effect of mental health stigma on disability outcomes in the same trend. The research showed that people who were undergoing stigma related to mental health disorders were more prone to disabilities and difficulties in reaching required facilities and support. This is because such individuals are unable to take enough measures to stay safe from disabling conditions as well as getting help to treat any ailments which could ultimately produce disability. This underlines the necessity of combating mental health stigma and creating an environment rich in supportive community to avert disability.

According to Arango et al.’s (10) study, individuals who have high mental well-being had a lower rate of disability compared to those with a low rate of mental well-being. Thus, such efforts are aimed at reducing the incidence of disability through programs meant to improve mental health and well-being. World Health Organization (11) studied the role of social support in the prevention of disability. The studies found that individuals with well-developed social networks are at a lower risk of being disabled compared to those with poor social networks. This stresses the role of social relationships in maintaining mental health and preventing any impairment. This is opposite to the individuals described in previous paragraph. Such individuals pay attention to the wellbeing of their physical health and seek medical and social support of resolve any preventable or curable issues in a timely manner.

Lastly, a study carried out by Petersen (12) focused on the effect of physical exercise on mental health and disability. As per the research, it was found that exercises done regularly were associated with low rates of impairment and better mental health. This implies that the physical exercise campaigns can be the cornerstone of the prevention campaigns for disabilities and enhancing mental health.

Overall, we can conclude that the mental health is linked with physical health. The factors which can ensure a good physical health are dependent on mental health such as physical activity, social interactions, recognizing health issues, seeking help from medical facilities, and treating diseases carefully. Same factors can be linked to disability prevention and management.

## 3 Methodology

This study’s approach consisted of a detailed narrative review of the body of literature that addresses the relationship between mental health and disability prevention.

### 3.1 Identification of relevant studies

The search was performed in online databases which include PubMed, Google Scholar, PsycINFO and Web of Science to get the relevant studies which were published in peer-reviewed journals. The keywords including mental health, preventive medicine, and wellbeing were used to lead the search. Keywords used in the search comprised ‘mental health promotion,’ ‘wellbeing,’ ‘disablement prevention,’ ‘mental health interventions’ and ‘wellbeing programs’. The search was restricted to articles in English, and which were published within the past eleven years. No specific geographic area was targeted. The reason to choose this duration was based on the relevant literature found. We have focused on such articles which describe a relationship or some links between mental health and disability. The authors recommend a future systemic review of the findings for a longer duration and draw a comparison among various findings.

### 3.2 Inclusion and exclusion standards

Articles included in the review addressed the issues of mental health meditation targeting disability and wellbeing promotion. The efficacy of interventions, programs, and strategies with the purpose of improving mental health and preventing disability was investigated in studies that were added to the review. After screening, 50 articles were added to the review after they met the inclusion requirements. The articles which were not providing a relationship between mental health and disability have been excluded. Additionally, articles not written in English were also excluded.

### 3.3 Data extraction

The research design, demographics, intervention specifications, and findings concerning the prevention of disabilities and mental health were extracted from the chosen studies. For identifying common themes and gaps in the literature related to enhancing mental health and welfare to prevent disability, the retrieved data were then synthesized and evaluated.

### 3.4 Analysis and synthesis of findings

The research results were analyzed and summarized by narrative synthesis. The analysis was conducted to determine themes and patterns of the connection between promoting mental health issues and avoiding disabilities.

### 3.5 Quality assessment

The studies included were assessed for quality by using the critical appraisal checklist from the Joanna Briggs Institute,^1^ when required. General inclusion and exclusion criteria are described above (13).

### 3.6 Limitations

It is essential to keep in mind that this review is based on the earlier works and might be biased due to languages, publishing, or other possible factors. The review is more limited in terms of knowledge level and accessibility because of the type of research included. New empirical studies need to be carried out to further explore the correlation between the prevention of disability and promotion of mental health in future studies.

## 4 Results and discussion

### 4.1 Understanding mental health and wellbeing

#### 4.1.1 Definition of mental health and wellbeing

The emotional and social welfare of an individual is the subject of mental health and wellness (14–16). It constitutes of people’s convictions, mental state, and behaviors as they encounter the problems of life, elaborate on them, makes decisions, and build relationships. Besides the absence of mental disorders, mental health is all about the presence of positive emotions, adaptability, resilience, and a sense of belonging in life (12). A wider scope of general happiness, life satisfaction, self-acceptance, and fulfillment in many different areas of life are reflected in well-being (17). Thus, mental health contributes to managing all aspects of a person’s life.

#### 4.1.2 Factors influencing mental health and wellbeing

A person’s mental health and wellbeing is the result of numerous factors. These include psychological factors like personality traits and biological factors like genetics, brain chemistry and stress-coping skills; the social factors like relationships and socio-economic status; and the environmental factors like living conditions, access to healthcare and exposure to stressors (18). As an illustration, Shastri (14) study demonstrated that social networks and happiness are positively correlated, implying that social ties play an important role in determining mental health and welfare. Apart from that, physical activity, nutrition, sleep, and substance use are also some of the other factors that can affect mental health status and well-being (19). As an example, regular exercise has been proven to raise mood, reduce stress and enhance self-confidence. The above is associated with improvement in mental health. Just the same, long-term stress, insufficiency of sleep, and unhealthful way of eating patterns may have a negative impact on mental health and become a mental problem (20). It is of utmost importance that we understand the fact that different people will have different factors contributing to their mental health. Understanding the numerous factors and identifying specific factors for different individuals can help to develop a patient-centered management plan.

#### 4.1.3 Consequences of poor mental health on disability

An individual’s capacity to complete daily activities may be severely restricted by poor mental health, which then leads to disability (21). Disorders of mental health such as schizophrenia, depression, and anxiety influence the way people reflect, come to a decision, and relate with others. Subsequently, an individual finds it challenging to associate with other people, get jobs or go to school. Statistics, Waddell and Burton (17) found that mental health disorders, when untreated, can lead to a higher incidence of social isolation, unemployment, and work-related disability.

Besides that, suboptimal mental health may add to the severity of physical health issues and bring about a chain of co-morbidities that increase the chance of disability. Untreated depression, for instance, can significantly reduce self-care behavior, thus contributing to poorer physical health outcomes increase in disability (17). In addition, the stigma associated with mental health problems may make people hesitant to seek help or get treatment, and this could aggravate their condition and worsen the long-term harm (22).

This is clear from the literature referred in this section that disturbance in mental health would create health problems which could ultimately result in disability. Thus, to prevent disability, it is essential to keep mental well-being which will influence healthy life.

### 4.2 The link between mental health and disability

#### 4.2.1 Relationship between mental health issues and disability

Mental health problems and disabilities are often mutually reinforcing since individuals suffering from mental health issues are more vulnerable to disabilities (23). There is growing evidence that psychological disorders like depression, anxiety, PTSD, and schizophrenia can greatly hinder daily living and prevent one from full function in society, thus causing disablements (24). It can be very hard for severe depression sufferers to keep their relationships, work, and even to take care of themselves. Consequently, they can develop functional disorders which fulfill the disabilities’ criteria. Additionally, if anxiety disorders are left untreated, they can result in avoidance behaviors and social isolation, thus limiting an individual’s effectiveness in numerous life spheres (25).

In addition, mental health stigma and judging compound the development of a disability by creating another barrier to supporting care or accommodations (26). Persons suffering from mental health problems can experience obstacles when it comes to getting employment, housing, education and healthcare that are essential for quality of life (27). The combination of these factors can result in a situation where mental health problems arise or become worse. Thus, a cycle of disability is created.

#### 4.2.2 Impact of mental health problems on the risk of disability

Numerous studies have demonstrated that diseases of mental health encourage a person’s risk of becoming disabled. For example, a longitudinal study by Nicholson (15) realized that those with severe mental health problems would face a high chance of lasting disabilities, such as limited mobility, doing tasks on your own and communicating with others. The study showed the complicated relationship between mental health and disabilities, as symptoms of psychiatric disorders could go all the way to functional disturbance and worsen the general contentment of an individual.

Furthermore, a few psychological disorders have been identified to be linked with cognitive impairment, a condition which may affect an individual’s ability to do normal tasks and keep their job (28). People with these diseases may be weak in the areas that require executive functioning, attention, and memory, which could create challenges as they struggle with organizing their thoughts, planning their time, and completing more complex tasks (29). Cognitive deficiencies entail marked limitations to autonomous functioning and heightened needs for external assistant devices or provisions; these can all lead to the label of disability (7). Moreover, people with mental health and physical health problems together bear a higher risk of developing physical disability, as having the two health problems at the same time can have an overwhelming impact on their wellbeing (30). To illustrate this, a patient with chronic pain and concomitant depression may have physical activity limitations, suffer from poor sleep quality, and be socially withdrawn more often; this could result in further development of debility (31). The simultaneous occurrence of some mental and physical health problems can make treatment and rehabilitation more complex, as individuals might have problems in complying with medication regimens, attending therapy sessions, or performing self-management behaviors necessary for maintaining healthy lifestyle (32).

Based on the literature discussed in this section, we can conclude that a good physical health, which acts as a barrier against disability development, is dependent and linked to mental health.

### 4.3 Strategies for promoting mental health and wellbeing

#### 4.3.1 Social support and network

Social support can be the enabler of mental health and safety, which will prevent disability occurrence (6). Even, such support is helpful in management and treatment of conditions which can produce permanent disability. People with strong social networks who get support from friends and members of the community may tend to have lower levels of stress and anxiety (33). For instance, Petersen (12) showed that social support is a protective shield that prevents anxiety from affecting mental well-being. Furthermore, Jané-Llopis et al. (34) points out how emotional and instrumental support could contribute to improving mental health. Enhancing social support and creating nurturing social networks can be achieved through interventions such as support groups, community engagement programs, and helping each other initiatives (35). These strategies will facilitate in building good relationships, connection to others, emotional support, and empathy during difficult times, thus impacting positively in individuals’ mental resilience and wellbeing (17). Overall, social support plays a role in prevention, management and treatment of ailments which ultimately can produce disability.

#### 4.3.2 Physical health and exercise

Exercise and physical fitness are two of the most powerful characteristics for mental and holistic well-being (36). Regular workouts have also been proven to reduce anxiety and depressive symptoms and improve general cognitive performance. In a meta-analysis done by Turcotte et al. (37), exercise was found to substantially influence mental health indicators, such as reduced risk of depression and improved quality of life. In addition, Thieme et al. (9) research suggests that physical activity may be useful alongside the prevention of mental illnesses. Incorporating regular exercise and physical activities into daily routines is a way of improving mental health and reducing risk of disability (38). Health promotion actions, like exercise programs, wellness course, and outdoor recreational sports, can persuade participants to become healthy and consider their wellness (39).

When they practice exercising on a regular basis, individuals can elevate their mood and have their mental toughness increased (2). A healthy state of body is not only a preventive factor for development of diseases but also helps in speedy recovery from developed ailments. This also includes conditions which might complicate to disabilities if not treated timely and properly.

#### 4.3.3 Psychological therapy and counseling

Psychotherapy and counseling constitute fundamental strategies of preventing disability and promoting mental health and emotional wellbeing (40). The efficacy of evidence-based treatment options has been shown to tackle many kinds of mental disorders. Examples of these therapies are mindfulness-based interventions and interpersonal therapy. For example, according to Curran et al. (41), CBT has proven effective in dealing with various psychological disorders.

Access to mental health services and psychological aid is essential for people suffering from mental health (4). From the standpoint of a community psychoeducation program, the availability of low cost counseling, therapy sessions, and mental health services can give people the motivation they need to seek help, deal with their emotional needs, and adopt survival tactics when dealing with stress and improve mental wellbeing (42). Through embedding psychological therapy and counseling into existing mental health promotion programs, individuals receive the support they need to ameliorate their emotional resilience and avert the occurrence of disabling mental health situations (6). These techniques help to build mental strength.

#### 4.3.4 Stress management techniques

The stress management techniques indeed are indispensable instruments for improving mental health and combating disability (43). Chronic stress has been linked to mental health conditions such as burnout, depression, and high anxiety (44). Through stress-reduction strategies such as mindfulness meditation, deep breathing, and cognitive reconstruction, one gets to understand stress and become more resilient to mental health issues (9). The research by Arango et al. (10) has demonstrated that practicing mindfulness-based stress reduction leads to less stress and better psychological well-being. In addition, the studies conducted by Petersen (12) have underscored the role of effective coping strategies in shielding mental health against the damaging effects of stress. Empowering people with the tools for stress management by integrating those techniques into mental health promotion can help an individual to deal effectively with stress and achieve a feeling of calm and balance and thereby their emotional well-being (39). Through stress reduction strategies and resilience-building techniques, people can build healthier coping mechanism, be less vulnerable to mental health problems and might possibly maintain a sound mind.

### 4.4 Impact on disability prevention

#### 4.4.1 Evidence supporting the link between mental health and disability

Several investigations have shown that mental health is closely related to disability (17, 19, 45). A person’s mental health has a significant role to play in their ability to cope with daily activities, leading to low productivity, impaired relationships, and high susceptibility to disability (23). For instance, a depression patient might find it hard to stay in a job owing to difficulties with focusing and decision making, which would consequently increase the chances of long-term disability.

A report by Corrigan et al. (46) established that the most severely mentally ill were more likely to experience disability than those who do not have any mental health problems. The research spotlighted that it is crucial to attend to mental health as a way of avoiding disability. Moreover, the study of Wahass (25) showed that people with no treatment for their mental health conditions were twice more likely to report functional restrictions and disabilities than those who were in treatment.

#### 4.4.2 Case studies or examples of successful mental health interventions

Several mentally healthy interventions have been put in place to creatively prevent disabilities due to mental disorders. For example, the “Healthy Minds” program of the WHO aims to incorporate mental health and resilience advocacy into community-driven activities (10). The program includes the element of psycho education, stress management techniques and social support strategies to improve mental health outcomes and decrease the risk of disability.

Large companies in different case studies have collaborated with mental health specialists and come up with mental health plans at workplace which improved employees mental health outcomes with a reduction in absenteeism and claims for disability (14). The program was comprised of frequent mental health screenings, available counseling services and some stress management workshops that all together led to better mental health outcomes for employees.

#### 4.4.3 Recommendations for policy makers and healthcare providers

Based on the evidence supporting the link between mental health and disability and successful interventions, several recommendations can be made for policy makers and healthcare providers:

Invest in mental health promotion and prevention programs: Policy makers should spend resources to develop and apply mental health promotion programs that focus on early intervention and prevention methods. Through the enhancement of mental health and well-being, the risk of disabilities may be lessened (16).

Incorporate mental health facilities into primary care settings: To achieve early detection and curing of mental health issues, the practitioners in health care need to include screening and treatments of mental health in primary care setups. Therefore, this may limit the progress of mental health problems that may be incapacitating later on (18).

Provide training and support for mental health professionals: Policymakers need to provide training programs especially for mental health specialists which aim to boost their skills in evidence-based mediation for those who are vulnerable to develop disabilities. Life-long professional development and support will enable mental health practitioners to be abreast with modern research and good practice that hinder mental health and enable disability (23).

Foster collaboration between healthcare providers and community organizations: It is advisable that stakeholders encourage interaction between healthcare personnel and employees to develop a multidimensional mechanism for tackling mental illness and preventing disability. They can jointly develop coordinated strategies to deal with different dimensions of mental health issues within the society (25).

The policies should be designed and directed toward various setups which play a role in mental and physical health management such as educational institutes, hospitals, mental health care providers, related government ministries, and community organizations involved in help and support individuals with mental health and/or disability.

### 4.5 Challenges and barriers

#### 4.5.1 Stigma surrounding mental health

The stigma of mental illness is regarded as the major obstacle to the practice of stopping disability by mental health and wellness promotion. Several studies have found that people who struggle with mental health disorders often experience prejudice and discrimination, resulting in low level of care. Per the study by Coiffait et al. (47) for example, those with mental disorders typically are looked at labeled as aggressive or incompetent and therefore keep all to themselves and end up isolated from society.

The effectiveness of prevention programs may be hindered because of this stigma; they may be avoided by people who are afraid of being judged or labeled (48). It is of paramount importance to enlighten the public and educate people about mental health conditions in order to fight this stigma. Through facilitating tolerance and knowledge of mental health problems, we will mitigate stigma and build a community where folks feel more comfortable seeking help. In addition to that, developing a positive attitude toward mental illness and breaking stereotypes can make access to resources easier for people and motivate them to do something when it comes to their mental health.

#### 4.5.2 Access to mental health services

Implementing mental health and well-being as a strategy for disability prevention still holds a lot of barriers that include access to mental health treatment. Studies have continued to prove that there is an inequality in access to health care, among the vulnerable groups such as the poor and those in less developed regions. For example, the researcher (49) found that compared to their white counterparts, people from minority backgrounds tend to underutilize mental health care because of a variety of barriers such as lack of insurance, difficulty in communicating in another language, and cultural stigma.

Making mental health services accessible requires a multi-pronged approach that includes increasing the availability of these services; addressing language and cultural challenges; and policies to provide equitable access to all. Promotion of care availability can also be greatly supported by telehealth and digital mental health programs, especially in rural or remote locations where traditional services may be difficult to access (7). People could get the help they need to avoid disability and improve general well-being by expanding mental health care coverage. Such services should also target early recognition of health issues which can produce disability in long run.

#### 4.5.3 Funding and resources for prevention programs

One of the main barriers to establishing mental health and welfare programs to prevent disability is the scarcity of funds and resources for prevention programs. Research has demonstrated that prevention programs are not always given priority in healthcare funding, creating gaps in services, and therefore limiting availability of evidence-based interventions. For instance, a recent (20) study established these prevention programs for mental health disorders are being underfunded by comparison to the treatment programs that offer more significant cost savings and benefits through early interventions.

The need for more financial and resource allocation for preventative programs that can develop mental health and wellness is crucial to solving this problem. Adding to the reduction of mental health related problems and long-term disability prevention can be made real through funding the preventive efforts (50). In addition, we need to focus on the efficiency of prevention programs and support strategies that encourage early intervention and comprehensive approaches to mental health promotion (51). By channeling appropriate resources and allocating enough money to prevention, we can build a more sustainable and efficient system for mental health promotion and disability minimization (24). Financial support is very important for smooth and effective running of various related programs and institutes.

### 4.6 Implications for policy and practice

#### 4.6.1 Policy implications for promoting mental health and preventing disability

This study’s findings suggested the need to include mental health promotion elements in preventative policies of disability. Mental health not only affects physical well-being but overall wellbeing too. Moreover, encouraging mental health can be useful in restricting the spread of mental health conditions that can progress into disability (26). Thus, the decision-makers need to include mental health promotion as the top priority in their disability prevention strategies. This might be achieved by establishing programs that bring to light the role of positive mental health, coping mechanisms or resilience. Prevention of disability is hugely influenced by policies that advocate for mental health awareness and which also provide access to mental health treatment services (29).

For example, McDaid (31) research found that teenagers who are offered mental health promotion programs at schools have fewer issues with mental health problems and they do better academically and generally. Our research demonstrated that maintaining mental health and preventing deterioration require early intervention and a preventive strategy.

#### 4.6.2 Strategies for integrating mental health promotion within existing disability prevention initiatives

Organizations and service providers can adopt various mental health promotion strategies and integrate them with present disability prevention initiatives. Another approach can be to educate the disability prevention professionals and health care providers on mental health awareness and counseling skills so that they are able to identify the mental health needs of those people who are at risk of disabilities (3). To facilitate the provision of wholesome and inclusive support services for those with mental and physical health challenges, mental health and disability services should collaborate.

Moreover, integrating mental health screening together with assessments for disability prevention in the regular screenings would help detect early signs of mental illnesses which might eventually cause disability (27). Through being proactive, treatments and support services can be designed to simultaneously fulfill the needs of mental and physical illness. Another strategy can be emphasizing self-care and well-being as part of disability prevention program. The promotion of individuals’ participation in doing activities that boost their well-being, such as mindfulness, relaxation techniques, and social support networks, can help in reducing stress, anxiety, and other mental health risks, which can prevent disability (41). By considering and managing mental health and disability under same umbrella could enhance the understanding and development of integrated strategies to manage.

#### 4.6.3 Recommendations for future research and practice

Going forward, it is imperative to conduct further research and practice that investigates the relationship between the promotion of mental health and the prevention of disability. Longitudinal studies that follow up on the mental health outcomes of people participating in the disability prevention programs can therefore be very informative regarding the effectiveness of the integrated interventions (46). Similarly, investigations on the socioeconomic determinants of mental health and disability may reveal gaps and provide a platform for the development of targeted interventions for the disadvantaged. Practitioners need to emphasize person-centered approaches that address the individual needs and preferences of people with specific diseases. Through involving citizens in planning and decision-making processes, service providers will be able to provide services that are individualized to address their own mental health and disability prevention needs (11).

Besides, initiatives to include mental health promotion within disability completion activities must be covered by funders and policy makers as well (33). The development of comprehensive and multidisciplinary service packages which meet the mental health needs can result in long-run cost savings and better outcomes for the individuals who are at risk of disability. Table 1 summarized the strategies, facilities, and measures linked with the discussion and description in this article.

**Table 1:** Strategies, facilities, and measures to improve mental health, disability prevention, and management.

Mental health awareness (community and related institutes) Disability awareness (community and related institutes) Disability and mental health relationship awareness campaigns Promoting stress management techniques Creating and promoting social support groups for mental health and/or disability Workplace programs for mental health Creating psychotherapy and counseling facilities Mental health programs at larger scales (e.g., policies promotion, implementation, and awareness at national level) Promoting physical activities, and their role in mental health and disability prevention and management Disability assessment and rehabilitation centers Hotlines and telehealth facilities for instant help and guidance Promoting and ensuring role of early intervention and collaborative care Linking various facilities to for a collaborative care team Facilitation in access to management facilities

## 5 Conclusion

In summary, providing mental health and a sense of wellbeing becomes a major factor to avoid disability. The review has shown the complexity of the relationship between mental health, wellness and disability which reinforces the importance of taking mental health issues seriously and addressing them at the community and national levels. By emphasizing inhibition and mediation aspect at early stage, we can reduce disablement and promote general wellness. It is essential that healthcare professionals, policymakers, and the community to make sure mental health promotion is a priority in all areas of healthcare and social services. It is important to understand that development of disability due to mental health issues would further deteriorate mental health as disturbance in quality due to disability would further increase stress. This vicious cycle needs to be stopped and prevented. We can only achieve this by understanding the importance of mental health and the wellbeing of every individual which in the long term will contribute to the creation of a healthier and more inclusive society.

## Footnotes

^1^ https://jbi.global/critical-appraisal-tools
